# Endoscopic Resection Versus Surgical Resection for Early Gastric Cancer: A Systematic Review and Meta-Analysis

**DOI:** 10.1097/MD.0000000000001649

**Published:** 2015-10-30

**Authors:** Weili Sun, Xiao Han, Siyuan Wu, Chuanhua Yang

**Affiliations:** From the Division of Gastroenterology and Hepatology, Ren Ji Hospital, School of Medicine, Shanghai Jiao Tong University, Shanghai Institute of Digestive Disease, Shanghai, China.

## Abstract

Endoscopic resection (ER) has been widely accepted to treat early gastric cancer (EGC) in place of surgical resection (SR). The aim of this meta-analysis was to conduct a comprehensive comparison between the two methods.

Four literature databases, including PubMed, Web of Science, the Cochrane Library, and EMBASE, were searched for studies that compared ER with SR to treat EGC. In this meta-analysis, primary and secondary endpoints were compared between the two groups. Primary endpoints included overall survival (OS), disease-specific survival (DSS), disease-free survival (DFS), and recurrence-free survival (RFS). Secondary endpoints included operation-related death, local recurrence, metachronous lesions, procedure-related complication, bleeding, hospital stay, operation time, and cost.

Nineteen studies consisting of a total of 6118 patients were identified and selected for evaluation. Meta-analysis showed that long-term outcomes of ER versus SR for EGC were comparable in terms of 5-year OS (risk ratio [RR] 1.00, 95% confidence interval [CI] 0.98–1.02), DSS (RR 0.98, 95% CI 0.89–1.08), DFS (RR 0.95, 95% CI 0.86–1.05), and RFS (RR 0.98, 95% CI 0.94–1.01). However, ER had shorter operation time (standardized mean difference [SMD] −3.39, 95% CI −3.58 to 3.20), hospital stay (SMD −2.86, 95% CI −4.02 to −1.69), lower costs (SMD −5.30, 95% CI −10.37 to −0.22), and fewer procedure-related complications (RR 0.43, 95% CI 0.28–0.65) compared to SR. Nevertheless, ER had higher incidences of local recurrence (risk difference 0.01, 95% CI 0.00–0.02) and metachronous lesions (RR 6.81, 95% CI 3.80–12.19).

Endoscopic resection was associated with similar long-term outcomes and considerable advantages concerning operation time, hospital stay, costs, and complications, compared with SR, and was also associated with disadvantages such as higher incidence of local recurrence and metachronous lesions. Further high-quality studies from more countries are required to confirm these results.

## Abstract 2

Supplemental Digital Content is available in the text

## INTRODUCTION

Early gastric cancer (EGC) is defined as a lesion or carcinoma that is limited to the mucosa or submucosa, regardless of lymph node involvement. Surgical resection (SR) with lymph node dissection is considered a conventional treatment for EGC, which can result in favorable long-term outcomes with a 5-year overall survival (OS) rate of ≥95%.^1^ However, owing to minimal invasiveness, low cost, faster recovery, and better quality of life after the procedure, endoscopic resection (ER) including endoscopic mucosal resection (EMR) and endoscopic submucosal dissection (ESD) has become widely accepted as a standard treatment for any EGC lesion defined as a differentiated intramucosal adenocarcinoma (≤2 cm in diameter) without submucosal extension and ulceration.^2,3^ Moreover, the development of endoscopic technology has allowed other lesions at negligible risk of lymph node metastasis (such as larger lesions, lesions with ulceration, and undifferentiated lesions) to be included in the expanded indications for ER.^4^ Several recent single-arm studies, investigating the efficacy of ESD for treating EGC cases meeting these expanded indications, have also demonstrated favorable short-term and long-term clinical outcomes.^5^ However, this method, despite its efficacy, sometimes has some major disadvantages including high incidence of metachronous gastric cancer.^6^ Moreover, very little is known about long-term clinical outcomes of EGC patients who have been treated with ER compared with those who have undergone SR. In recent years, several long-term follow-up studies have compared ER with SR for EGC.^7–10^ However, the results of these studies were not entirely consistent, and limited definite conclusions were reached considering the safety and effectiveness of these two methods. To date, there has not been any meta-analysis conducted that has combined data from studies that compared the outcomes of ER and SR for EGC treatment. We therefore conducted a meta-analysis of eligible studies to compare the efficacy and safety of ER and SR among EGC patients.

## MATERIALS AND METHODS

This systematic review and meta-analysis was conducted in line with Preferred Reporting Items for Systematic Reviews and Meta-Analyses (PRISMA). Ethical approval and patient consent were not necessary because this study is a “Systematic Review and Meta-Analysis.”

### Search Strategies

We initially performed a systematic literature search in Pubmed, EMBASE, Web of Science, and the Cochrane Library through May 20, 2015, to identify eligible articles that compared ER with SR for treatment of EGC. There were no language restrictions. The searching keywords were: “endoscopic resection,” “endoscopic mucosal resection,” “endoscopic submucosal dissection,” “surgical resection,” “gastrectomy,” “surgery,” and “early gastric cancer.” The reference bibliographies of eligible studies and review articles were screened manually for other possible studies.

### Study Selection

The inclusion criteria for eligible studies include the following: EGC diagnosis confirmed by histology test; studies compared the efficacy and safety between ER and SR for EGC. For studies based on data from the same population, only one with high quality was included. Any studies presented as case reports, review articles, commentaries, editorials, and letters were excluded. Studies that did not provide the outcomes of interest were also excluded.

### Data Extraction and Study Quality Assessment

The information extracted from the eligible studies was as follows: first author, year of publication, country, study duration, endoscopic procedure, type of surgery, number of patients (ER/SR), mean age, and the endpoints. For those studies that were excluded because it used the same data as another, information was also extracted to identify whether supplementary information existed. Two investigators (W.L.S. and X.H.) independently performed the data extraction and reached a consensus on discrepant items through discussion. As all included studies were nonrandomized studies, the Newcastle-Ottawa Scale (NOS) was adopted to assess the methodological quality.^11^ Studies that obtained scores of ≥7 were considered as high-quality studies.

### Evaluation Criteria for Endpoints

Primary endpoints were as follows: OS—the proportion of patients who had survived from any causes of death after ER or SR; disease-specific survival (DSS)—the proportion of patients who had survived from only gastric cancer related death; disease-free survival (DFS)—the proportion of patients who had survived without gastric cancer recurrence, occurrence of a new gastric cancer, or death of any cause since ER or SR had been conducted; and recurrence-free survival (RFS)—the proportion of patients who had survived without tumor recurrence, death with evidence of recurrence, or occurrence of a metachronous gastric cancer after ER or SR.

Secondary endpoints were as follows: local recurrence—cancer diagnosed by histology within the previous ER scar or anastomosis sites during follow-up; operation-related death—death within 30 days after ER or SR; metachronous lesions—newly developed gastric cancers after 1 year of ER or SR; hospital stay—the period from the date of ER or SR to the discharge date; procedure-related complication—all complications during or after the operation; bleeding—bleeding during or after the operation; operation time—from marking to resection of the tumor; and cost—total cost of hospitalization during the treatment.

### Statistical Analysis and Data Synthesis

Considering that OS, DSS, DFS, and RFS are time-to-event outcomes, hazard ratios (HRs) should be our first choice to calculate the overall estimates of effect in the meta-analysis. However, the reporting of HRs in the eligible studies was mostly poor. Therefore, the survival data as risk ratios (RRs) at the 3 and 5-year marks were presented in our study. When possible, RRs at the 10-year mark were also represented. In some trials, only Kaplan–Meier survival curves were provided, and time-to-event outcomes were estimated using the method as described by Parmar et al.^12^ In addition, mean and variance were estimated using methods as described by Cochrane Book or Hozo et al,^13,14^ when the type of continuous date was presented as median and range. RRs with 95% confidence intervals (CIs) were also recommended for dichotomous data, such as procedure-related complications, bleeding, and metachronous lesions. The risk difference (RD) was adopted to evaluate operation-related death and local recurrence, due to the possibility of no death or local recurrence occurring in either group. The standardized mean difference (SMD) was recommended for continuous data, such as hospital stay, operation time, and cost. The chi-square and *I*^2^ statistics were applied to determine the statistical heterogeneity between pooled studies. *P* < 0.05 or *I*^2^ > 50% was considered as significant heterogeneity. A random-effects model was used when significant heterogeneity was detected between studies, whereas a fixed-effects model was applied when there was no statistical heterogeneity between studies. In addition, sensitivity and subgroup analysis were conducted to assess the stability of the results and to investigate the sources of heterogeneity, according to age of patients (aged ≥65 years), study quality (≥7 scores), studies published after the year 2010 (year 2010), different endoscopic procedures (EMR and ESD), different indications for use of ER (absolute and expanded), and sample size of study (≥200). Funnel plots were performed to assess publication biases. RevMan 5.2 software (Cochrane Collaboration, London, UK) was used in our meta-analysis. Results with *P* value less than 0.05 were considered statistically significant.

## RESULTS

### Search Results and Study Selection

**FIGURE 1:**
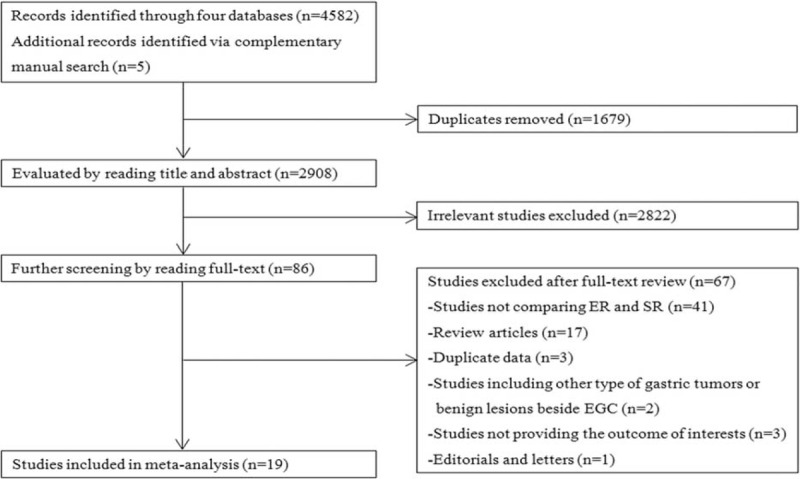
Flow chart for article screening. EGC = early gastric cancer, ER = endoscopic resection, SR = surgical resection.

A total of 4587 potential studies were generated through our search strategy. Eighty-six potentially appropriate articles were selected for further screening after excluding duplicates and unrelated studies. Upon the full text review, 67 studies were excluded for the following reasons: 41 did not compare ER and SR; 18 were editorials and reviews; 3 studies did not provide the outcomes of interest; 2 studies included other types of gastric tumors or benign lesions apart from EGC; and 3 studies were published on the basis of the same data. Thus, the remaining 19 studies^7–10,15–29^ (15 full text articles and 4 abstracts), including 3871 patients in the ER group and 2247 patients in the SR group, were eligible for the meta-analysis. All included studies were nonrandomized controlled trials and were conducted in Asian countries, including China, Japan, and Korea. Four studies^8,25,27,28^ compared ER with SR in elderly patients (aged ≥65 years) with EGC. Ten studies^7–10,15,16,23–26^ included in the meta-analysis were considered to be of high quality according the NOS score. The process of our study selection is showed in Figure 1. The key characteristics of the eligible studies are presented in Table 1.

**TABLE 1:**
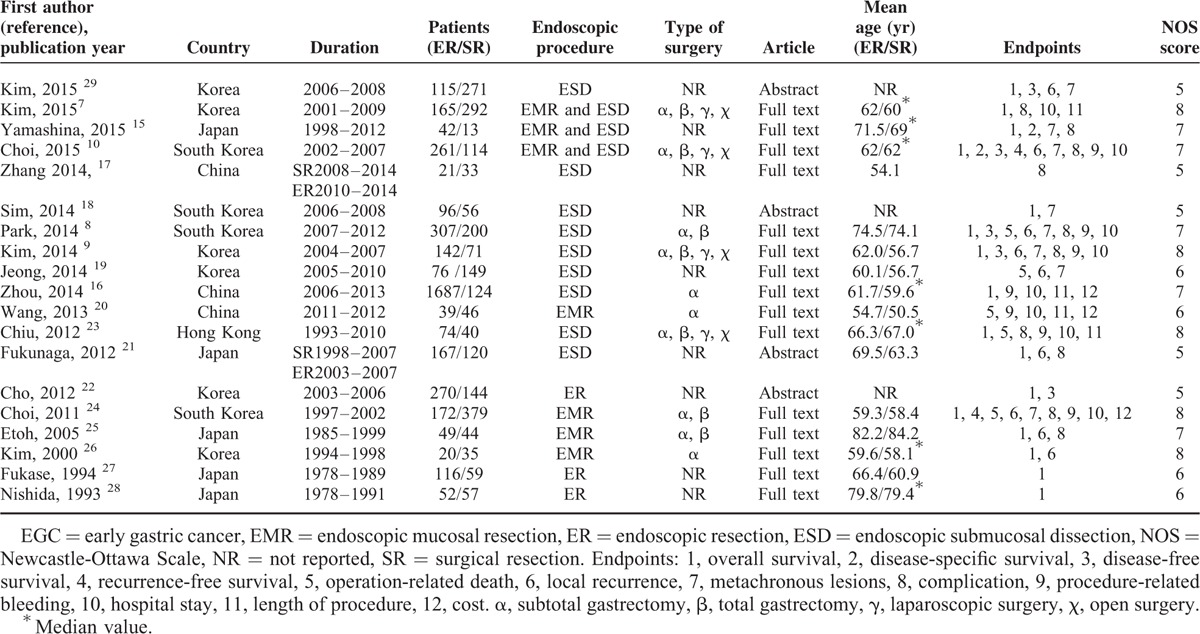
Characteristics of Eligible Studies

### Overall Survival and Disease-Specific Survival Rates

**FIGURE 2:**
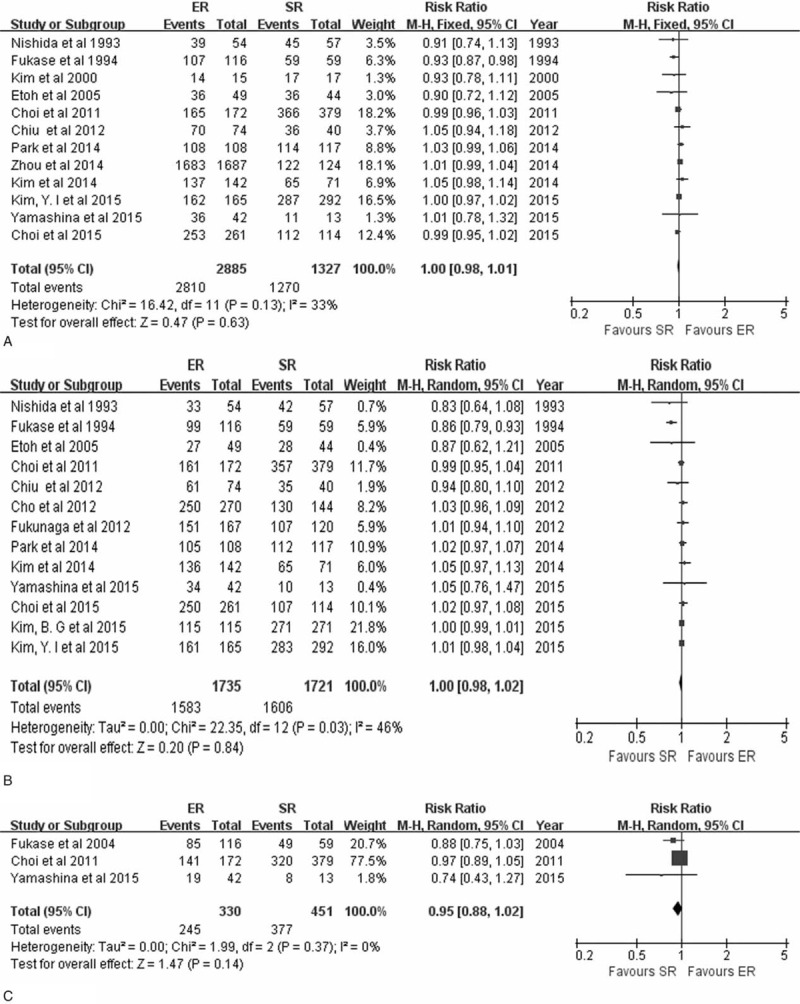
Forest plots of 3-year overall survival rate (A), 5-year overall survival rate (B), 10-year overall survival rate (C). CI = confidence interval, df = degrees of freedom, ER = endoscopic resection, M-H = Mantel-Haenszel, SR = surgical resection.

The 3, 5, and 10-year OS rates were obtained in 12 studies,^7–10,15,16,23–28^ 13 studies,^7–10,15,21–25,27–29^ and 3 studies,^15,24,27^ respectively. Meta-analyses of all these studies revealed no statistically significant difference in OS between ER and SR after 3 years (RR 1.00, 95% CI 0.98–1.01, *P* = 0.63), 5 years (RR 1.00, 95% CI 0.98–1.02, *P* = 0.84), and 10 years (RR 0.94, 95% CI 0.87–1.01, *P* = 0.10) (Figure 2).

**FIGURE 3:**
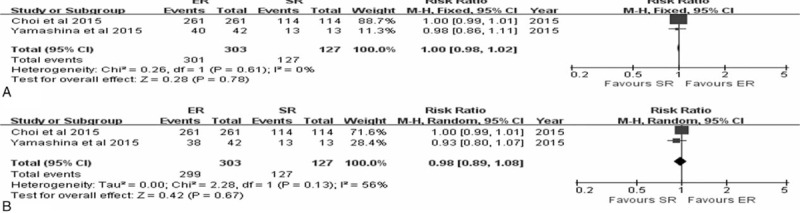
Forest plots of 3-year disease-specific survival rate (A), 5-year disease-specific survival rate (B). CI = confidence interval, df = degrees of freedom, ER = endoscopic resection, M-H = Mantel-Haenszel, SR = surgical resection.

The DSS was reported in two studies.^10,15^ Meta-analysis of the 3 and 5-year DSS of ER versus SR in these two studies revealed no statistically significant difference with a pooled RR of 1.00 (95% CI 0.98–1.02, *P* = 0.78) and 0.98 (95% CI 0.89–1.08, *P* = 0.67) on the basis of fixed-effects and random-effects model, whereby these models were used according to heterogeneity (*P* = 0.61, *I*^2^ = 0%; *P* = 0.13, *I*^2^ = 56%, respectively) (Figure 3).

### Disease-Free Survival and Recurrence-Free Survival Rates

**FIGURE 4:**
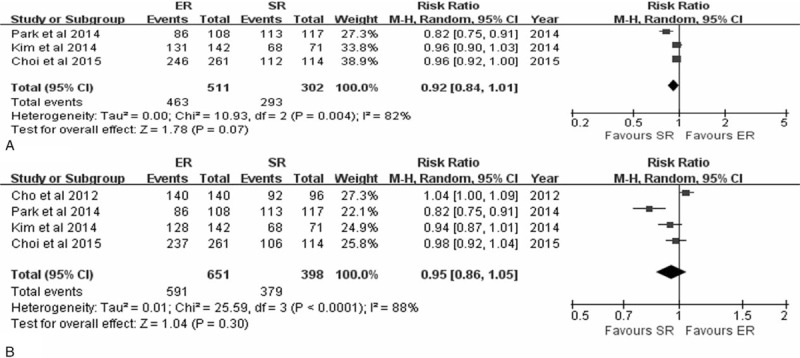
Forest plots of 3-year disease-free survival rate (A), 5-year disease-free survival rate (B). CI = confidence interval, df = degrees of freedom, ER = endoscopic resection, M-H = Mantel-Haenszel, SR = surgical resection.

The 3 and 5-year DFS was reported for EGC in three^8–10^ and four^8–10,22^ studies, respectively. The meta-analysis of the 3 and 5-year DFS of ER versus SR showed no statistically significant difference, with pooled RRs of 0.92 (95% CI 0.84–1.01, *P* = 0.07) and 0.95 (95% CI 0.86–1.05, *P* = 0.30), based on a random-effects model due to significant interstudy heterogeneity (*P* = 0.004, *I*^2^ = 82%; *P* *<* 0.0001, *I*^2^ = 88%, respectively) (Figure 4).

**FIGURE 5:**
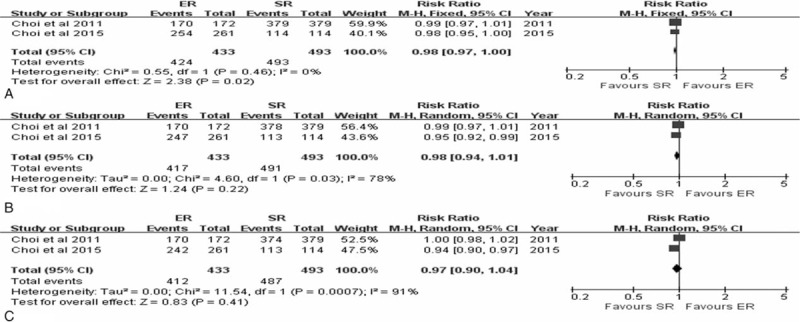
Forest plots of 3-year recurrence-free survival rate (A), 5-year recurrence-free survival rate (B) and 10-year recurrence-free survival rate (C). CI = confidence interval, df = degrees of freedom, ER = endoscopic resection, M-H = Mantel-Haenszel, SR = surgical resection.

The RFS for EGC was described in two studies.^10,24^ Pooling the data of these studies revealed a lower 3-year RFS with ER in comparison with SR (RR 0.98, 95% CI 0.97–1.00, *P* = 0.02), with no interstudy heterogeneity (*P* = 0.46, *I*^2^ = 0%). However, the pooled analysis of the 5 and 10-year RFS of ER versus SR revealed no statistically significant difference, with pooled RRs of 0.98 (95% CI 0.94–1.01, *P* = 0.22) and 0.97 (95% CI 0.90–1.04, *P* = 0.41) based on random-effects model due to statistical heterogeneity (*P* = 0.03, *I*^2^ = 78%; *P* = 0.0007, *I*^2^ = 91%, respectively) (Figure 5).

### Procedure-Related Complication and Mortality

**FIGURE 6:**
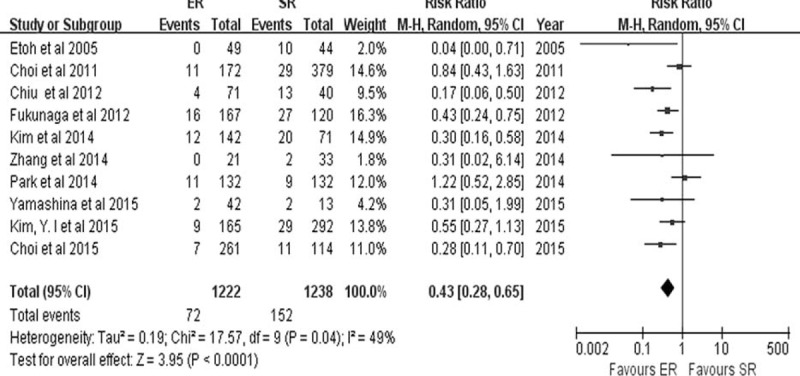
Forest plots of procedure-related complication. CI, confidence interval; df = degrees of freedom, ER = endoscopic resection, M-H = Mantel-Haenszel, SR = surgical resection.

**FIGURE 7:**
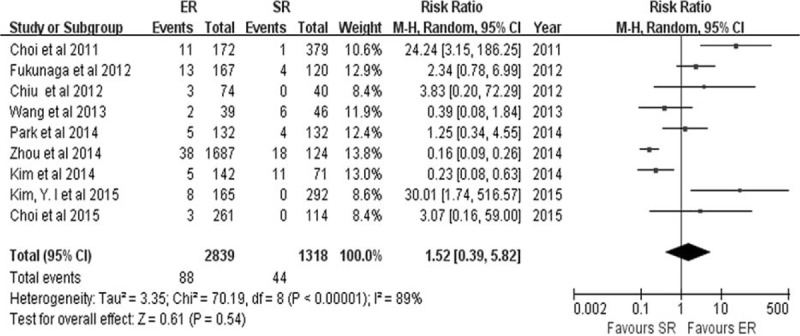
Forest plots of procedure-related bleeding. CI = confidence interval = df, degrees of freedom, ER = endoscopic resection, M-H = Mantel-Haenszel, SR = surgical resection.

The majority of studies only reported major complications, such as bleeding, perforation, intestinal obstruction, anastomotic leakage, and postoperative adhesion, which needed further interventions including endoscopic treatment or reoperation. The rate of procedure-related complications, from 10 relevant combined studies, was lower in the ER group (72/1222) than in the SR group (152/1238) (RR 0.43, 95% CI 0.28–0.65, *P* < 0.0001).^7–10,15,17,21,23–25^ (Figure 6). Statistical heterogeneity was observed among the studies (*P* = 0.04, *I*^2^ = 49%), and a random-effects model was used. Procedure-related bleeding was reported in 9 studies.^7–10,16,20,21,23,24^ The pooled analysis of the bleeding rate showed no statistically significant difference between the ER group (88/2839) and the SR group (44/1318) (RR 1.52, 95% CI 0.39–5.82, *P* = 0.63) (Figure 7), and statistical heterogeneity was detected between the studies (*P* < 0.0001, *I*^2^ = 89%). As one of the most common complications of ER, perforation was reported in 9 studies.^7–10,15,16,21,23,25^ The perforation rate was 1.11% (52/4700) in the ER group.

**FIGURE 8:**
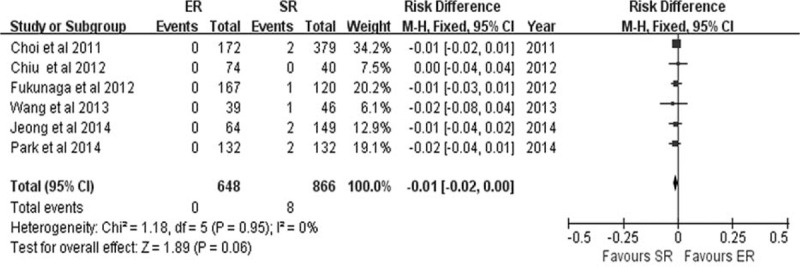
Forest plots of operation-related death. CI = confidence interval, df = degrees of freedom, ER = endoscopic resection, M-H = Mantel-Haenszel, SR = surgical resection.

Six studies^8,19–21,23,24^ described procedure-related death. The rate of procedure-related death was lower in the ER group (0/648) than in the SR group (8/866), but no statistically significant difference was found (RD −0.01, 95% CI −0.02 to 0.00, *P* = 0.06) (Figure 8). No heterogeneity was observed among the studies (*P* = 0.95, *I*^2^ = 0.0%).

### Local Recurrence and Metachronous Lesions

**FIGURE 9:**
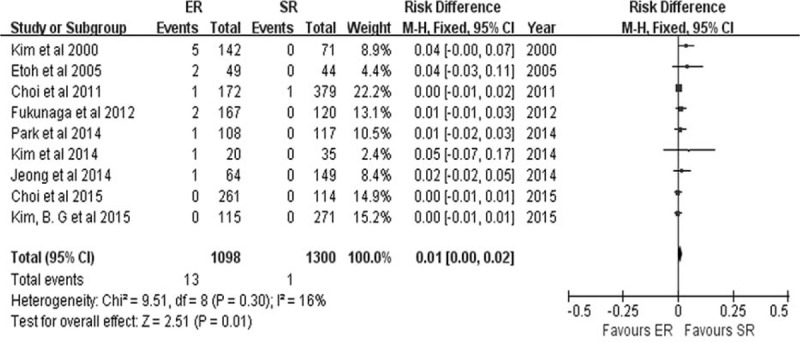
Forest plots of local recurrence rate. CI = confidence interval, df = degrees of freedom, ER = endoscopic resection, M-H = Mantel-Haenszel, SR = surgical resection.

Local recurrence was reported in 9 studies.^8–10,19,21,24–26,29^ The local recurrence rate was higher in the ER group (13/1098) than in the SR group (1/1300) (RD 0.01, 95% CI 0.00–0.02, *P* = 0.01) (Figure 9), and no heterogeneity was detected among the studies (*P* = 0.30, *I*^2^ = 16%).

**FIGURE 10:**
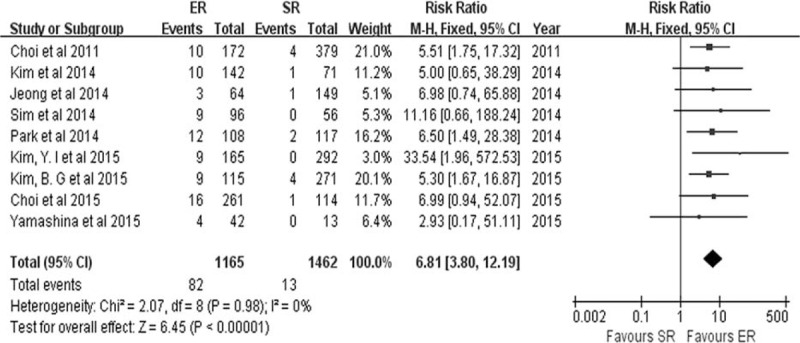
Forest plots of metachronous lesions. CI = confidence interval, df = degrees of freedom, ER = endoscopic resection, M-H = Mantel-Haenszel, SR = surgical resection.

Nine studies^7–10,15,18,19,24,29^ described the development of metachronous lesions after ER and SR for EGC. The incidence of metachronous lesions was higher in the ER group (82/1165) than in the SR group (13/1462) (RR 6.81, 95% CI 3.80–12.19, *P* < 0.0001) (Figure 10), and there was no heterogeneity among the studies (*P* = 0.98, *I*^2^ = 0.0%).

### Hospital Stay, Operation Time and Cost

**FIGURE 11:**
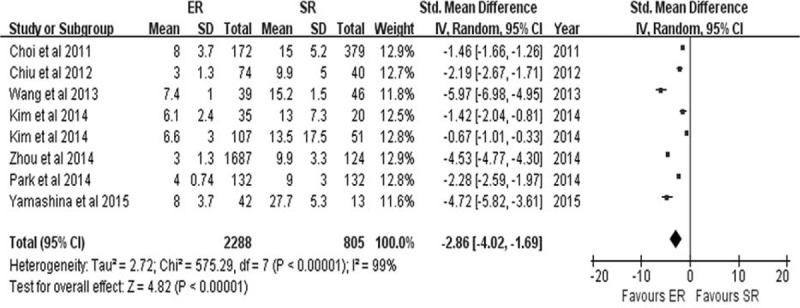
Forest plots of hospital stay. CI = confidence interval, df = degrees of freedom, ER = endoscopic resection, M-H = Mantel-Haenszel, SD = standard deviation, SR = surgical resection.

**FIGURE 12:**
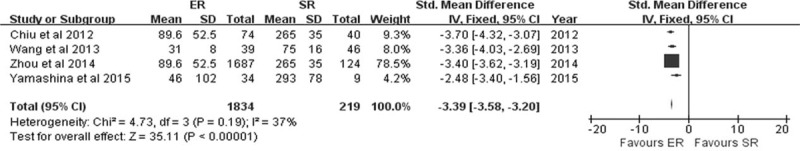
Forest plots of operation time. CI = confidence interval, df = degrees of freedom, ER = endoscopic resection, M-H = Mantel-Haenszel, SD = standard deviation, SR = surgical resection.

**FIGURE 13:**
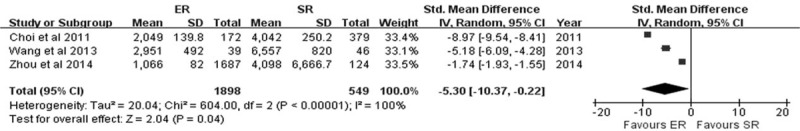
Forest plots of cost. CI = confidence interval, df = degrees of freedom, ER = endoscopic resection, M-H = Mantel-Haenszel, SD = standard deviation, SR = surgical resection.

There were 7,^8,9,15,16,20,23,24^ 4,^15,16,20,23^ and 3^16,20,24^ studies that reported the hospital stay, operation time, and cost of ER versus that of SR for EGC, respectively. Shorter hospital stay, operation time, and lower cost were observed in the ER group than in the SR group, with a pooled SMD of −2.86 (95% CI −4.02 to −1.69, *P* < 0.0001) (Figure 11); −3.39 (95% CI −3.38 to −3.20, *P* < 0.0001) (Figure 12); and −5.30 (95% CI −10.37 to −0.22, *P* = 0.04) (Figure 13), respectively. Statistical heterogeneity was detected between the pooled studies on hospital stay and cost (*P* < 0.0001, *I*^2^ = 99%; *P* < 0.0001, *I*^2^ = 100%, respectively), and a random-effects model was used.

### Sensitivity and Subgroup Analyses

Sensitivity and subgroup analyses are shown in Table 2. There were no significant differences in the 3 and 5-year OS rates between the two groups, in any of the subgroups. Lower rates of procedure-related complications and higher incidence of local recurrence with ER compared to SR were found in all subgroups, although statistical significance was not reached in all the subgroups. This may due to the small number of studies included in the analysis.

**TABLE 2:**
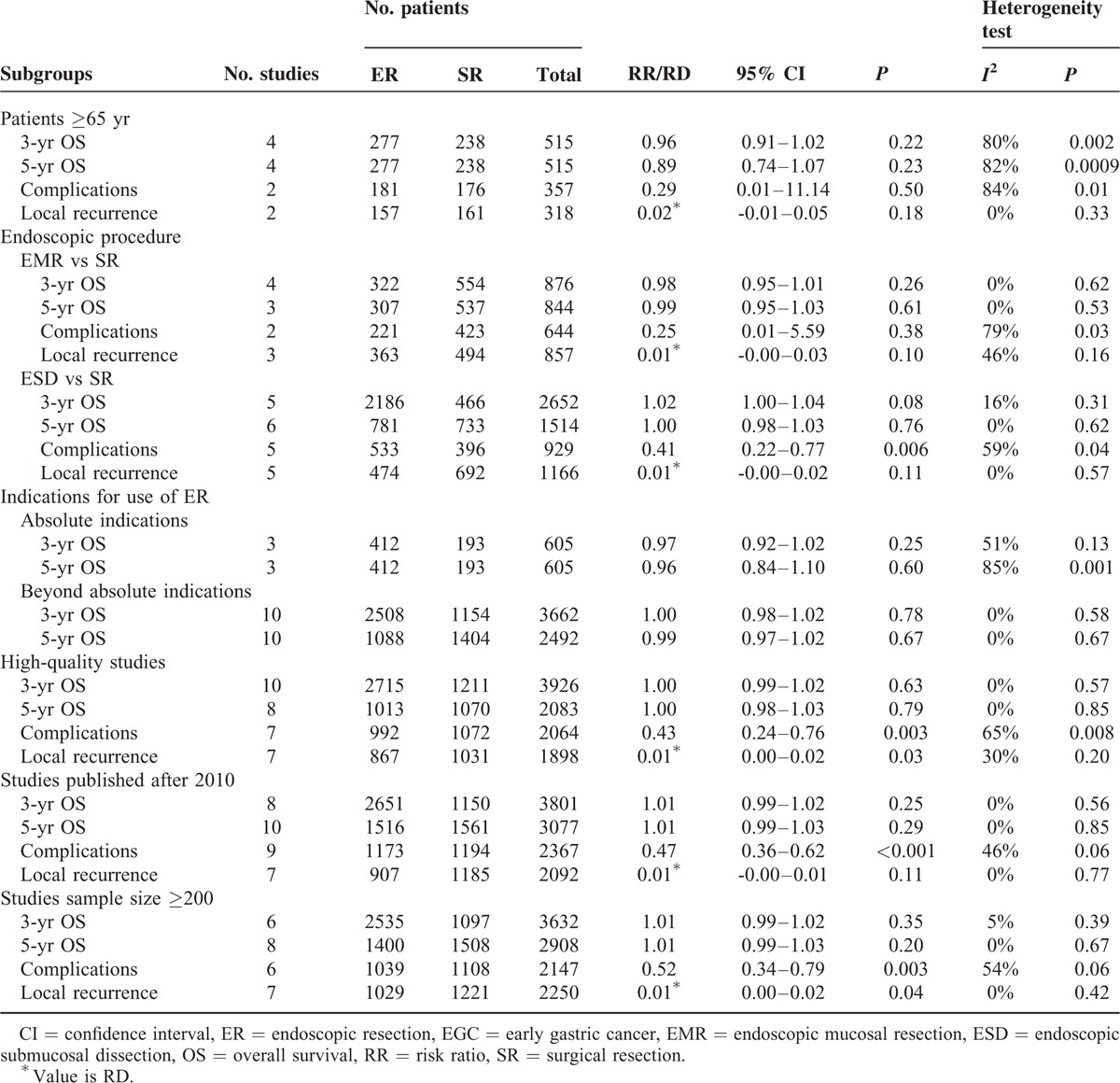
Results of Sensitivity and Subgroup Analyses

On the basis of funnel plot, no publication bias was found when we adopted the 5-year OS as the outcome (Supplementary Figure 1, http://links.lww.com/MD/A440).

## DISCUSSION

The current meta-analysis demonstrated that long-term outcomes of ER versus SR for treatment of EGC were comparable in terms of OS, DSS, DFS, and RFS. However, ER had shorter operation time and hospital stay compared to SR. Lower procedure-related costs and rate of procedure-related complications were also observed in the ER group. However, ER was associated with a higher incidence of local recurrence and metachronous lesions.

Endoscopic resection which was developed in Japan has been increasingly considered as an acceptable therapeutic option for EGC, with no concomitant lymph node metastasis worldwide, due to its minimal invasiveness and low cost.^30^ Long-term outcomes, such as OS and DFS, were the most valued factors for the EGC patients. This meta-analysis has determined that the long-term outcomes were comparable between ER and SR. There are some available explanations for the results. Firstly, selection of ER in the treatment of EGC strictly follows indications conforming to the absolute or expanded criteria. In addition, with the development of endoscopic technology, ESD can offer a higher rate of en bloc resection and complete resection compared to other conventional endoscopic treatments, and tends to be chosen for EGC treatment.^31^ Moreover, despite ER being associated with a higher incidence of local recurrence and metachronous gastric cancer compared to SR, careful follow-up surveillance after ER plays a great role in detecting these gastric cancers at an early stage, and most of them can be successfully treated by repeated ER.^32^ Furthermore, several studies discussing surgical training in ESD for treatment of EGC have shown that ESD can be performed after the experience of 30–60 procedures, and those procedures performed by supervised residents can achieve similar complete resection rate and complication rate when compared to experienced endoscopists.^33,34,35^

Many recent studies about the efficacy of ESD on EGC, which meet the expanded indication criteria, have reported favorable short-term and long-term clinical outcomes.^5^ In one study, the comparable long-term outcomes of ESD were demonstrated when compared with SR, according to the absolute and expanded criteria, respectively.^9^ A meta-analysis exploring the outcome of ESD for treatment of EGC of absolute and expanded indications revealed that the long-term outcome of patients in absolute indications group was comparable to that of patients in expanded indications group.^36^ In our meta-analysis, only 2 studies^10,27^ strictly complied with the absolute criteria, whereas the other 13 studies were beyond the absolute criteria. However, the long-term outcomes (5-year OS) of ER were still comparable to those of SR (RR 0.99, 95% CI 0.97–1.02) when only studies that used expanded criteria were included. Thus, it is reasonable to recommend ESD to be widely adopted for the lesions within expanded indications.

Little is known about the clinical and long-term outcomes of ER in elderly patients. In the present meta-analysis, 4 of the included studies compared the clinical outcomes of ER with SR in elderly patients (aged ≥65 years). When combining these studies, comparable long-term outcomes regarding the 3-year OS (RR 0.96, 95% CI 0.91–1.02) and 5-year OS (RR 0.89, 95% CI 0.74–1.07) between the two groups were determined. Complication rate was lower in the ER group (RR 0.29, 95% CI 0.01–11.14), but with no statistical significance, perhaps due to just 2 studies being included. In addition, 1 study about clinical safety of ESD and surgery in EGC patients aged ≥70 years reported that long-term survival rates in the absolute indication group showed no difference from those in the group beyond the absolute indication, but within the expanded indication.^8^ The expanded criteria of ER for EGC may therefore also apply to elderly patients. However, the study also reported the development of metachronous lesions was more frequent in ESD patients (12/108) compared to surgery patients (2/117). As such, ER should be recommended as an initial treatment for elderly EGC patients on the basis of careful follow-up surveillance to detect local recurrence or metachronous lesions after ER.

The advantages of ER compared with SR for EGC were operation time, hospital stay, cost, and procedure-related complication, and its major drawbacks were the high rate of local recurrence and metachronous lesions development. ER is minimally invasive and preserves the whole stomach, which can increase the risk of metachronous cancers on unresected parts of the stomach compared to SR. Furthermore, it was reported that metachronous cancers often develop in the middle or lower-third of the stomach where the majority of the primary gastric cancers tend to occur.^8^ Nakajima et al^37^ showed that the overall incidence of metachronous gastric cancers after ER was 8.20% and the annual incidence was constant. Our results were consistent with previous studies. In our study, the incidence of metachronous lesions was 7.04% (82/1165) in the ER group compared to 0.89% (13/1462) in the SR group. However, although the incidence of local recurrence and metachronous gastric cancer was higher in the ER group, most of them could be detected by periodic endoscopic surveillance at an early stage and curatively treated by repeated ER. In addition, a meta-analysis showed that the eradication of *Helicobacter pylori* in patients who have undergone ER for EGC could also reduce the occurrence of metachronous gastric cancer.^38^

The procedure-related complication rates of ER and SR in our meta-analysis were 5.89% (72/1222) and 12.28% (152/1238), respectively. SR was associated with more frequent major complications, such as bleeding, intestinal obstruction, anastomotic leakage, anastomosis site stricture, ischemic or perforated viscera, and postoperative adhesion, which needed further interventions including endoscopic treatment or reoperation and resulted in high expenditure and long-term hospitalization. Compared with SR, the major complications of ER were less frequent and largely manifested as bleeding and perforation. In our study, the perforation rate associated with ER was approximately 1.11% and the bleeding rate was 3.10%. It has been suggested that bleeding or perforation complications after ER in patients with EGC can be successfully managed by endoscopic treatment. The procedure-related mortality was 0.92% in the SR group and 0.00% in the ER group in the meta-analysis. Although statistical significance was not reached, a higher rate of mortality was associated with SR.

There are several limitations in the present study. Firstly, no randomized controlled trials were included in our meta-analysis, and baseline characteristics of patients in the two groups were not rigorously matched in some of the eligible studies. Therefore, the quality of the included studies may have an influence on the results which presented an advantage for ER. Secondly, explicit and complete definition or information of some items, such as the definition of the cost, operation time, and specific figures of some long-term outcomes, were not provided in certain studies. It is therefore difficult to extract accurate data for meta-analysis, and as such, the results may be affected. Thirdly, a very small number of studies provided data on particular endpoint components, such as DFS and RFS, and the results based on these components may therefore have been somewhat underpowered. Fourthly, there was significant interstudy heterogeneity in several of the analyses. Fifthly, different indications for ER and several types of endoscopic therapies and surgeries were used. There were differences in treatment efficacy between each modality. This could raise an important bias in the meta-analysis. Finally, all the studies included in the current meta-analysis were conducted in Asia; however, more studies (including those carried out in the West) must be included in further analyses to confirm our findings.

In conclusion, compared with SR for EGC treatment, ER was associated with similar long-term outcomes and considerable advantages concerning procedure-related complications, operation time, hospital stay, and cost, but was also associated with disadvantages such as higher incidence of local recurrence and metachronous lesions. Further high-quality studies from more countries are required to confirm these results.
